# Low dose DMSO treatment induces oligomerization and accelerates aggregation of α-synuclein

**DOI:** 10.1038/s41598-022-07706-2

**Published:** 2022-03-08

**Authors:** Lasse Reimer, Caroline Haikal, Hjalte Gram, Vasileios Theologidis, Gergo Kovacs, Harm Ruesink, Andreas Baun, Janni Nielsen, Daniel Erik Otzen, Jia-Yi Li, Poul Henning Jensen

**Affiliations:** 1 Danish Research Institute of Translational Neuroscience - DANDRITE, Aarhus University, Aarhus C, Denmark; 2 Department of Biomedicine, Aarhus University, Aarhus C, Denmark; 3 Neural Plasticity and Repair Unit, Wallenberg Neuroscience Center, Department of Experimental Medical Science, Lund University, Lund, Sweden; 4 Interdisciplinary Nanoscience Center - iNANO, Aarhus University, Aarhus C, Denmark; 5 Institute of Health Sciences, China Medical University, 110112 Shenyang, People’s Republic of China

**Keywords:** Neuroscience, Molecular neuroscience

## Abstract

Dimethyl sulfoxide (DMSO) is a highly utilized small molecule that serves many purposes in scientific research. DMSO offers unique polar, aprotic and amphiphilic features, which makes it an ideal solvent for a wide variety of both polar and nonpolar molecules. Furthermore, DMSO is often used as a cryoprotectant in cell-based research. However, recent reports suggest that DMSO, even at low concentration, might interfere with important cellular processes, and cause macromolecular changes to proteins where a shift from α-helical to β-sheet structure can be observed. To investigate how DMSO might influence current research, we assessed biochemical and cellular impacts of DMSO treatment on the structure of the aggregation-prone protein α-synuclein, which plays a central role in the etiology of Parkinson’s disease, and other brain-related disorders, collectively termed the synucleinopathies. Here, we found that addition of DMSO increased the particle-size of α-synuclein, and accelerated the formation of seeding-potent fibrils in a dose-dependent manner*.* These fibrils made in the presence of DMSO were indistinguishable from fibrils made in pure PBS, when assessed by proteolytic digestion, cytotoxic profile and their ability to seed cellular aggregation of α-synuclein. Moreover, as evident through binding to the MJFR-14-6-4-2 antibody, which preferentially recognizes aggregated forms of α-synuclein, and a bimolecular fluorescence complementation assay, cells exposed to DMSO experienced increased aggregation of α-synuclein. However, no observable α-synuclein abnormalities nor differences in neuronal survival were detected after oral DMSO-treatment in either C57BL/6- or α-synuclein transgenic F28 mice. In summary, we demonstrate that low concentrations of DMSO makes α-synuclein susceptible to undergo aggregation both in vitro and in cells. This may affect experimental outcomes when studying α-synuclein in the presence of DMSO, and should call for careful consideration when such experiments are planned.

## Introduction

α-synuclein (α-syn) is central in the pathogenicity of the subgroup of neurodegenerative disorders collectively designated the synucleinopathies; a subgroup comprised of Parkinson’s disease (PD), Dementia with Lewy Bodies (DLB) and Multiple System Atrophy (MSA). α-syn itself presents as a 140-amino acid protein that is widely expressed in the brain, where it primarily localizes to the presynaptic terminals in neurons. The native structure of α‑syn is still debated, but a majority of reports suggest, that under physiological conditions, α‑syn exists mainly as an intrinsically disordered monomer^1,2^. However, tetramerization of α-syn under physiological conditions has been proposed^3,4^. Besides α-syn, two other synuclein family members exist, namely β- and γ-synuclein. While γ-synuclein primarily resides in the peripheral nervous system, α- and β-synuclein (β-syn) exhibit comparable brain-specific expression patterns^5^. Yet, structurally, β-syn diverges from α-syn within the non-amyloid-β component (NAC) domain indispensable for α-syn aggregation^5^, which is considerably truncated in β-syn. Consequently, β-syn shows much lower tendency to aggregate^6^.

Under pathological conditions α-syn aggregates into larger insoluble species, but α-syn is also hypothesized to exist as intermediate structures, referred to as oligomers, in between the native monomeric and insoluble fibrillary states. Such soluble α-syn oligomer species have been detected in post-mortem PD, DLB and MSA patient brain extracts^7–9^, and accumulating evidence suggest direct participation of oligomers in disease progression in the brain of patients suffering from synucleinopathies^10–12^. Based on the hypothesis that aggregation of α‑syn constitutes the major decisive event in the development of pathology, several intervention strategies to prevent oligomerization, including immunization- and small molecule based strategies, have been developed. Furthermore, a number of tools to visualize and assess the size and structure of α-syn, e.g. conformation specific antibodies exist.

In the current study, we investigate how dimethyl sulfoxide (DMSO), a widely utilized polar aprotic solvent, affects α-syn structure and aggregation. DMSO is often used to dissolve water-insoluble organic and inorganic compounds, including compounds associated with modulatory effects on α-syn aggregation as well as chemical cross-linkers used to stabilize tetrameric cellular α-syn^3,4,13^. Although a few reports mention an effect of DMSO treatment on α-syn structure^14,15^ the effect of DMSO in experiments has generally been neglected.

Here, we demonstrate that low concentrations of DMSO induces a shift in size-distribution of α-syn into larger species. Furthermore, incubation of monomeric α-syn with DMSO gives rise to enhanced binding to the MJFR-14-6-4-2 antibody, which preferentially recognizes aggregated forms of α-syn, and accelerates the rate of fibrillation without affecting the overall structure and seeding properties of the fibrils. Simultaneously, cells exposed to DMSO exhibit higher levels of α-syn multimerization as well as MJFR-14-6-4-2-positive inclusion-like structures. By contrast, no clear pathological changes were observed in α-syn structures in wt C57BL/6 or α-syn transgenic mice orally treated with DMSO for two weeks. Together, our findings suggest that when interpreting α-syn aggregation-specific data in experiments where DMSO is included caution should be taken: especially when working with α-syn in vitro and in cellulo.

## Results

### DMSO treatment increases α-syn particle-size and stimulates in vitro aggregation

**Figure 1:**
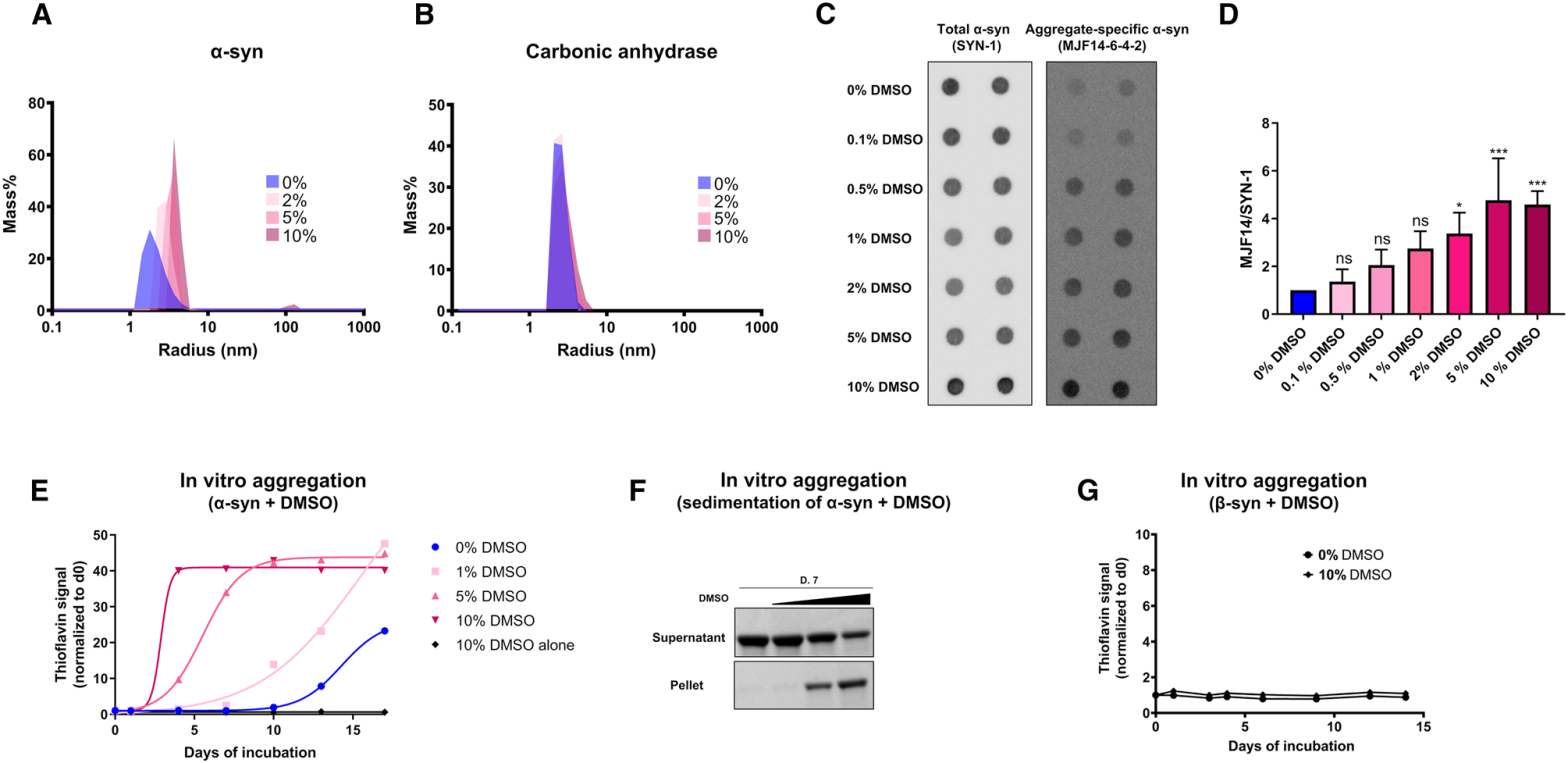
DMSO increases α-syn particle-size, aggregation-dependent antibody recognition and stimulates fibrillation (**A** and **B)** Dynamic light scattering analysis of α-syn (0.5 mg/mL, 35 µM) and carbonic anhydrase (0.5 mg/mL, 17 µM) incubated for 1 h with increasing concentrations of DMSO (0%, 2%, 5% and 10%). The figure demonstrates a mass% distribution based on the scattering intensity, with log scaled hydrodynamic radius depicted on the X-axis. Representative figure of three independent replicates (n = 3). (**C**) Dot blot of 100 ng of α-syn protein. Prior to loading, recombinant α-syn was incubated for 30 min at room temperature (RT) under non-agitating conditions in a concentration of 10 µg/mL (0.69 µM) in PBS alone or in combination with indicated concentrations of DMSO. α-syn was visualized using total α-syn (SYN-1) or MJFR-14–6-4–2 antibodies. (**D**) Quantification of MJFR-14–6-4–2/SYN-1 α-syn signal ratio normalized to 0% DMSO (n = 3, *p < 0.05, ***p < 0.001, one-way ANOVA followed by Dunnett’s multiple comparison test). (**E**) Lyophilized recombinant α-syn was re-suspended in sterile PBS alone (0% DMSO) or in combination with increasing amounts of DMSO (1%, 5% or 10%). A sample with 10% DMSO alone was included as negative control. The samples were incubated at 37 °C and 1050 rpm in a final α-syn concentration of 0.5 mg/mL (35 µM). ThT measurements were performed on 10 µl samples added to 100 μl of 40 μM ThT (final concentration) and signal measured (excitation at 450 nm and emission at 486 nm). Values were normalized to day 0 measurements for each sample and fitted as a sigmoidal growth curve. Representative figure of three independent replicates (n = 3). (**F**) Sedimentation assay of samples depicted in E) after 7 days of incubation. Equal volumes of each sample (0%, 1%, 5% and 10% DMSO) were pelleted by 25.000×*g* centrifugation for 30 min at 20 °C. The pellets were re-suspended and resolved on SDS-PAGE and stained using Coommassie Brilliant Blue. Depicted supernatant and pellet gel stainings were developed on two individual SDS-PAGE gels (n = 3). (**G**) Lyophilized recombinant β-syn was re-suspended in sterile PBS alone (0% DMSO) or in combination with 10% DMSO. The samples were incubated at 37 °C and 1050 rpm in a final β-syn concentration of 0.5 mg/mL (35 µM). ThT measurements were performed on 10 µl samples (excitation at 450 nm and emission at 486 nm), and normalized to day 0 measurements. Representative figure of three independent replicates (n = 3).

During our work with DMSO solubilized chemical cross-linkers, often used to verify the existence of cellular α-syn tetramers, we discovered an unexpected DMSO-dependent effect on α-syn structure. Combined with the widespread usage of DMSO in biomedical research, and limited but concerning information regarding the influence of DMSO treatment on α-syn behavior^14,15^, we decided to perform a comprehensive study*,* to elucidate the role of DMSO treatment on α-syn assembly. First, to assess the size distribution of recombinant α-syn in the absence and presence of low doses of DMSO, we performed a series of dynamic light scattering (DLS) analyses in the presence or absence of DMSO. Here, short exposure of α-syn to DMSO increased the particle size of α-syn in a concentration-dependent manner as evident both by mass% (Fig. 1A) and intensity% (Supplementary Fig. 1A). Contrary to the observations made with α-syn, DMSO did not induce a size shift in the globular control protein carbonic anhydrase (Fig, 1B and Supplementary Fig. 1B).

Based on these findings, we sought to examine if the increased particle size observed for α-syn could be caused by aggregation. Utilizing the MJFR-14-6-4-2 antibody which preferentially recognizes oligomeric and fibrillar forms of α-syn (Supplementary Fig. 2)^16^, we performed dot-blots on recombinant α-syn treated with different concentrations of DMSO. To control for equal loading between samples we used the SYN-1 antibody, which measures total α-syn levels (Fig. 1C). Contrary to SYN-1 detection, the MJFR-14-6-4-2 signal was significantly enhanced by DMSO treatment, suggesting that DMSO facilitates a structural change in α-syn which shares antibody recognition with that of aggregated α-syn present in neuronal inclusions in brain tissue (Fig. 1C and 1D)^17^.

Next, to assess the effect of the DMSO treatment on fibrillation, we used Thioflavin T (ThT) to measure β-sheet content in recombinant α-syn incubated at low levels in PBS, with increasing concentrations of DMSO under agitating conditions. We observed that the ThT signal of α-syn, incubated in the presence of 10%, 5% and 1% DMSO, increased and reached plateau intensity after two, 10 and 17 days of incubation respectively (Fig. 1E). By contrast, α-syn incubated in the absence of DMSO did not plateau after 17 days of incubation (Fig. 1E). Sedimentation of samples after 7 days of incubation, confirmed the presence of insoluble aggregates only in samples exposed to 5% and 10% of DMSO (Fig. 1F). By contrast, we did not observe any increase in ThT signal of β-syn, when exposed to DMSO (Fig. 1G). Taken together, these experiments suggest that short exposure to DMSO, even at low concentrations (< 2%), induces a size shift of α-syn, and promotes MJFR-14-6-42 antibody recognition (Fig. 1A and D), and while the α-syn fibrillation rate also increases by the addition of DMSO in vitro (Fig. 1E), insoluble aggregates are only formed after days of incubation at aggregation-promoting conditions (Fig. 1F).

### Pre-formed fibrils made in the presence of DMSO resemble naïve fibrils in structure, cytotoxicity profile and seed competence

**Figure 2:**
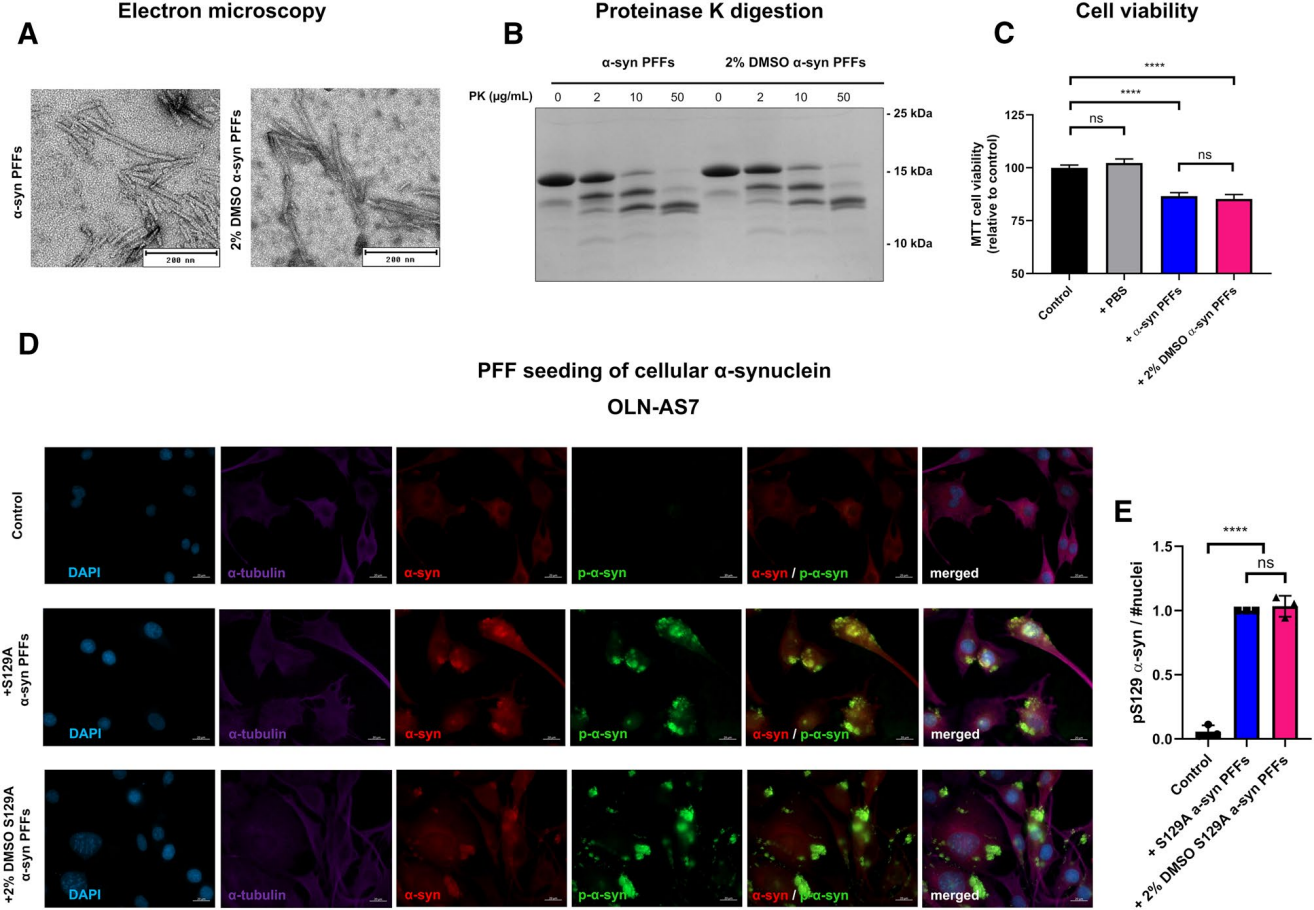
DMSO stimulated fibrils resemble naïve α-syn fibrils and maintain seeding capabilities (**A**) Representative image of TEM shows structure of wt α-syn PFFs or α-syn DMSO PFFs made in the presence of 2% DMSO. Scalebar = 200 nm. Representative images of three replicates (n = 3). (**B**) Digest samples of 25.000×*g* pelleted wt α-syn- and α-syn DMSO PFFs. Samples were digested with depicted concentrations of Proteinase K (PK). Digested samples were resolved on SDS-PAGE and stained at RT, using Coommassie Brilliant Blue. Representative gel of three independent replicates (n = 3). (**C**) Viability of α-syn-expressing SHSY5Y ASYN cells was measured by MTT assay 8 days post seeding. On day 2 the cells were left untreated (control) or treated with PBS (+ PBS), α-syn PFFs (28 μg/mL/2 μM) or 2% DMSO α-syn PFFs (28 μg/mL/2 μM) which was removed on day 4. Bars represent relative viability normalized to control. Three biological replicates with 10 measurements in each experiment (n = 3, ****p < 0.0001, one-way ANOVA followed by Tukey’s multiple comparison test) (**D**) Human a-syn expressing OLN-AS7 cells were exposed to PBS (Control) or 14 μg/mL (1 μM) sonicated S129A α-syn PFFs prepared in PBS alone (S129A α-syn PFFs) or in the presence of 2% DMSO (2% DMSO S129A α-syn PFFs). After 24 h of PFF treatment, the cells were washed to remove excess PFF and subsequently incubated for another 24 h before being fixed and visualized using DAPI (blue), α-tubulin (purple), total α-syn (red) or anti-phospho-S129 α-syn (green). Scale bar = 20 µm. Representative images of three biological replicates with 10 measurements in each experiment. (**E**) Quantification of area of anti-phospho-S129 α-syn signal relative to number of DAPI stained nuclei. Data are shown as mean of three independent experiments (n = 3, ****p < 0.0001, one-way ANOVA followed by Tukey’s multiple comparison test).

To evaluate the structure of the ThT positive and insoluble α-syn we examined in vitro aggregated α-syn by transmission electron microscopy (TEM) (Fig. 2A). Here, α-syn PFFs prepared in PBS alone (α-syn PFFs) and α-syn PFFs prepared in the presence of 2% DMSO (2% DMSO α-syn PFFs) both present as straight fibrils of comparable width and a similar overall appearance (Fig. 2A).

To further evaluate if the addition of DMSO to the aggregation process creates a distinguishable fibrillar polymorph of α-syn we performed a proteolytic peptide mapping of wt α-syn- and α-syn DMSO pre-formed fibrils (PFFs) and compared their proteolytic fingerprints: a technique that has been used extensively to determine structural differences between strains of α-syn^18–20^. Based on digests performed on purified PFFs with increasing concentrations of the serine protease, Proteinase K (PK), we evaluated the cleavage pattern (Fig. 2B). Here we observed that increasing amounts of PK, as expected, created gradually more and more degraded α-syn products (Fig. 2B). Yet, between the two groups of fibrils, an identical cleavage pattern was observed (Fig. 2B), demonstrating that DMSO stimulates α-syn aggregation without affecting the enzymatic accessibility of the PFFs. Furthermore, to test the cytotoxicity profile of the fibrils, human neuroblastoma SH-SY5Y cells, which by the addition of retinoic acid, readily differentiate into a non-mitotic phenotype where early developmental features of neurons are mimicked^21^, were treated with the two groups of PFFs. In this experiment we used a Tet-Off inducible variant (SH-SY5Y ASYN)^22^ of the cell-line where α-syn overexpression is induced by the removal of doxycyclin (dox). Here, we observed an equal loss in cell viability across treatment with 2 μM of the two PFF variants, while PBS treatment in itself appeared harmless (Fig. 2C).

Finally, to test the seeding potential of DMSO induced fibrils, we treated human α-syn-overexpressing OLN-AS7 cells, with α-syn PFFs to induce aggregation of cellular expressed α-syn. This cell-line has previously been successfully used to assess α-syn strain differences^23^. Again we used PFFs that were formed in PBS alone or induced by 2% DMSO. Yet, to distinguish between externally added PFF seeds, and the cellular expressed α-syn, we prepared PFF made from two distinct types of α-syn monomer, either those from monomeric wt α-syn or from S129A-substituted α-syn (S129A PFF) which cannot be phosphorylated on the S129 residue. First we evaluated how 1 μM of 2% DMSO S129A PFFs and regular S129A PFFs affected aggregation by assessing the cellular phospho-Ser129 (pSer129 α-syn) signal (Fig. 2D). Both DMSO- and regular S129A PFFs were able to induce an equally strong pSer129 α-syn signal, much higher than the background phosphorylation present in these cells (Fig. 2E). The effect on aggregation of both of these PFFs was further confirmed, using the MJFR-14-6-4-2 α-syn antibody (Supplementary Fig. 3A and B). To ensure that PFFs made from the S129A α-syn variant did not perturb the data, we also assessed pSer129 α-syn in cells treated with 2% DMSO PFFs and regular PFFs made from wt α-syn monomers. Again, treatment with 1 μM of PFFs induced a pSer129 α-syn signal highly increased from background, yet indistinguishable between the DMSO-induced and regular PFFs (Supplementary Fig. 4A and B). When we assessed the morphology of the PFF-induced aggregates in more detail, we noted that both the pSer129 α-syn- and MJFR-14-6-4-2 signal shared a strong co-localization pattern with that of total α-syn (Fig. 2D, Supplementary Fig. 3A and Supplementary Fig. 4A). However, both the pSer129 α-syn- and the MJFR-14-6-4-2 staining appeared more puncta-like compared to the diffuse total α-syn signal, suggesting the presence of inclusion-like α-syn structures. Together, these data demonstrate that α-syn aggregates stimulated by DMSO possess seeding-potential, and exhibit functional characteristics resembling those of naïve PFFs.

### DMSO stimulates α-syn multimerization in cells

Small molecules used to modulate cellular pathways are often dissolved in DMSO prior to experimental application. Although these compounds are most frequently diluted manifold upon the addition to cells the final molar concentration of DMSO can still be relatively high. Based on our in vitro findings we therefore investigated whether DMSO in its often-used concentration of 0.1–1%^24,25^ causes any significant changes in cellular α-syn structure.

**Figure 3:**
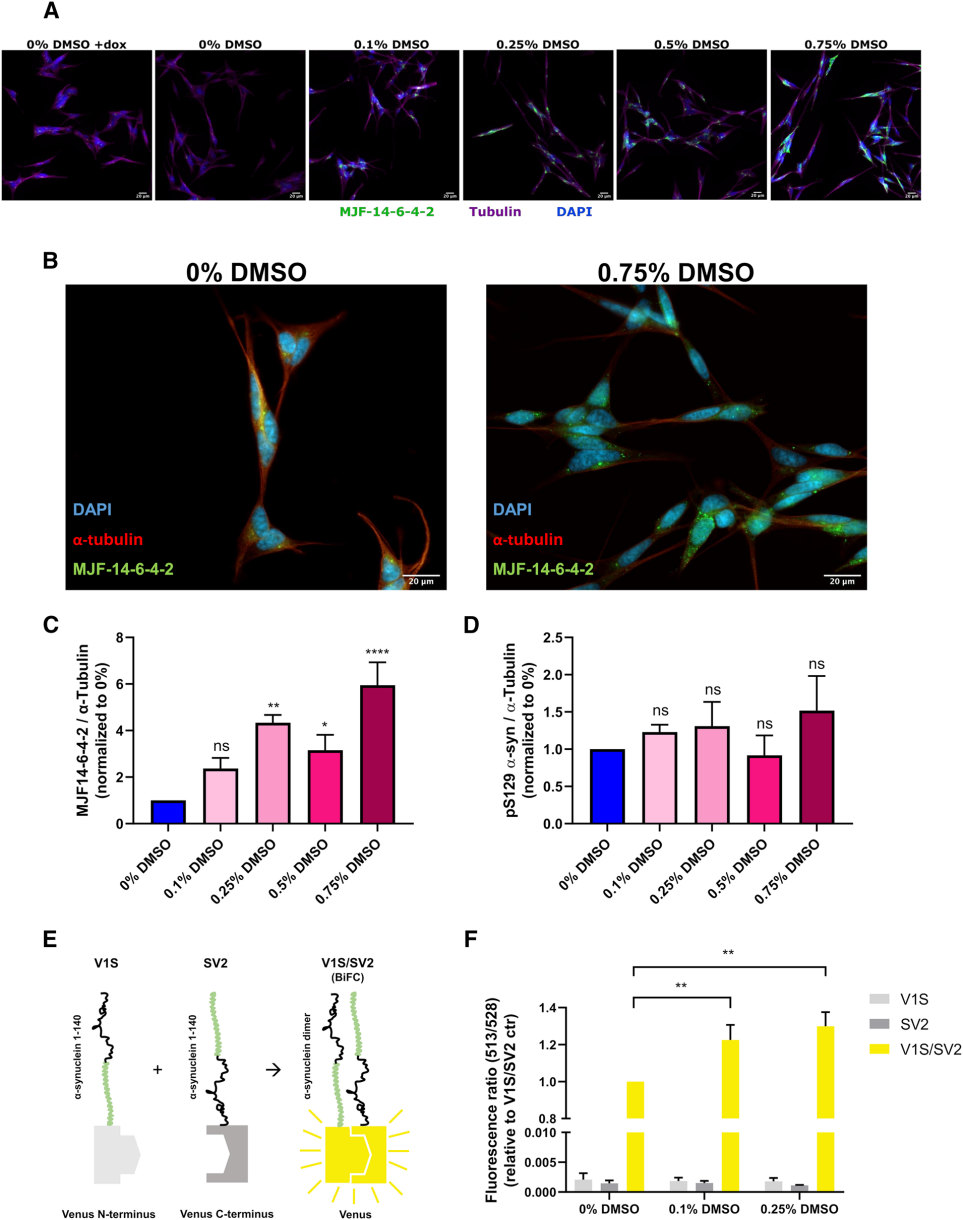
DMSO treatment induces α-syn multimerization in cells (**A**) SH-SY5Y ASYN cells were treated with dox to suppress α-syn expression (- α-syn) or relieved from dox treatment to induce α-syn overexpression. Cells relieved from dox were treated with increasing amounts of DMSO (0%, 0.1%, 0.25%, 0.5% or 0.75%) for 7 days prior to fixation, and visualized using DAPI (blue), MJFR-14–6-4–2 (green) or α-tubulin (purple). To stop mitosis, the SH-SY5Y ASYN cells were treated with retinoic acid (10 μM final concentration) during the experiment. Scalebar = 20 µm. (**B**) High magnification image of α-syn expressing SH-SY5Y ASYN cells treated with 0%- (left) or 0.75% (right) of DMSO for 7 days prior to fixation, and visualization using DAPI (blue), MJFR-14–6-4–2 (green) and α-tubulin (red). Scalebar = 20 µm. (**C**) Quantification of area of MJFR-14–6-4–2 signal from individual coverslips relative to area of α-Tubulin signal in α-syn expressing cells treated with increasing amounts of DMSO (0%, 0.1%, 0.25%, 0.5% or 0.75%) for 7 days (n = 4, with ~ 10 images for each condition in each experiment, *p < 0.05, **p < 0.01, ****p < 0.0001, one-way ANOVA followed by Dunnett’s multiple comparison test). (**D**) Quantification of area of anti-phospho-S129 α-syn signal from individual coverslips relative to area of α-Tubulin in α-syn expressing cells treated with increasing amounts of DMSO (0%, 0.1%, 0.25%, 0.5% or 0.75%) for 7 days (n = 3 with ~ 8 images for each condition in each experiment, one-way ANOVA followed by Dunnett’s multiple comparison test). (**E**) Explanatory figure of principle behind bimolecular fluorescence complementation (BiFC) assay using the fluorescent venus YFP construct. Upon correct dimerization of α-syn within the two fluorescent complementation pairs, V1S and SV2 will bring, the N- and C-terminus fluorescent fragments of Venus YFP within proximity, thereby allowing emission of its yellow fluorescent signal. (**F**) Quantification of YFP signal (excitation at 513 nm and emission at 528 nm) from Venus-α-syn oligomerization in non-treated and 0.1%- and 0.25% DMSO treated HEK-293 cells (20 h. treatment). Signal was normalized to non-treated V1S/SV2-expressing cells. (n = 3 with 10 measurements in each experiment, **p < 0.01, one-way ANOVA followed by Dunnett’s multiple comparison test).

When utilizing the human neuroblastoma SH-SY5Y ASYN cell line, we observed that α-syn overexpression, compared to α-syn non-overexpression cells, yielded a detectable baseline MJFR-14-6-4-2-, and phospho-Ser129 α-syn-specific staining after 7 days of differentiation (Supplementary Fig. 5A and B). Additionally, the MJFR-14-6-4-2 staining was significantly enhanced in cells continually treated with doses of DMSO that exceeded 0.25% (Fig. 3A–C), but already at 0.1% a trend was observed. Interestingly, the MJFR-14-6-4-2 staining morphologically appeared as small inclusions present in both the perinuclear region and in the processes within these cells (Fig. 3B). Contrary to the MJFR-14-6-4-2-staining, we did not observe elevated levels of phospho-Ser129 α-syn-specific staining in cells treated with DMSO at any of the applied concentrations (Fig. 3D).

As an orthogonal approach to the aggregate detection using MJFR-14-6-4-2 antibody, we assessed α-syn oligomerization in cells using an bimolecular fluorescence complementation (BiFC) assay with a split fluorescent venus YFP construct (Fig. 3E-F)^26,27^. This construct consists of two non-fluorescent pairs, namely V1S (N-terminal half of Venus YFP fused to N-terminus of full-length α-syn) and SV2 (C-terminal half of Venus YFP fused to C-terminus of full-length α-syn) that upon approximation reconstitute the intact fluorescent Venus structure. C to N- terminal dimerization of α-syn associated with V1S- and SV2 will bring the N- and C-terminal fluorescent fragments of Venus YFP within proximity, thereby allowing emission of its yellow fluorescent signal (Fig. 3E). Expression of these fluorescent complementation pairs in HEK-293 T cells, allowed evaluation of live α-syn multimerization within cells (Fig. 3F). To assess the role of low dose DMSO treatment on α-syn oligomerization we incubated the transfected cells with or without DMSO for 20 h prior to fluorescent analysis. Here we observed that even at low doses of 0.1% and 0.25% DMSO, equivalent to 1:1000 and 1:400 dilution respectively, induced an increase in α-syn multimerization based on YFP fluorescence (Fig. 3F). As expected, expression of individual V1S and SV2 construct did not induce YFP fluorescence in the presence or absence of DMSO (Fig. 3F).

Together, these cell-experiments suggest that DMSO treatment not only induce detectable α-syn aggregation-like species under in vitro experimental conditions, but also in a more complex cellular environment.

### Oral DMSO treatment does not induce gastrointestinal- or brain α-syn abnormalities in mice

Misfolding of α-syn in the enteric nervous system has been proposed to precede visible α-syn brain abnormalities, suggesting a model where PD pathology originating from the gut can spread to the central nervous system in a prion-like manner^28,29^. The initial site of aggregation in the gut is unknown, but α-syn-expressing endocrine cells that line the intestinal epithelium and connect to enteric neurons could be the site of initiation^30,31^. These cells are only separated by their plasma membrane from the gut lumen that may contains environmental toxins, pathogens and metabolites that could affect and initiate α-syn aggregation.

Based on this hypothesis we designed an experiment wherein wt C57BL/6, or transgenic F28 mice overexpressing human α-syn under the control of the endogenous mouse α-syn promoter^32^, were orally treated once a day with water, 10% DMSO (in a volume corresponding to 1 g/kg bodyweight) or 30% DMSO (in a volume corresponding to 3 g/kg bodyweight) for 14 days. Mice were subsequently sacrificed, and α-syn structures in the gastrointestinal tract and brain analyzed by immunohistochemistry (IHC) using the MJFR-14-6-4-2 antibody, which in cells proved effective in detecting enhanced α-syn aggregation upon DMSO treatment (Fig. 3A–C).

**Figure 4:**
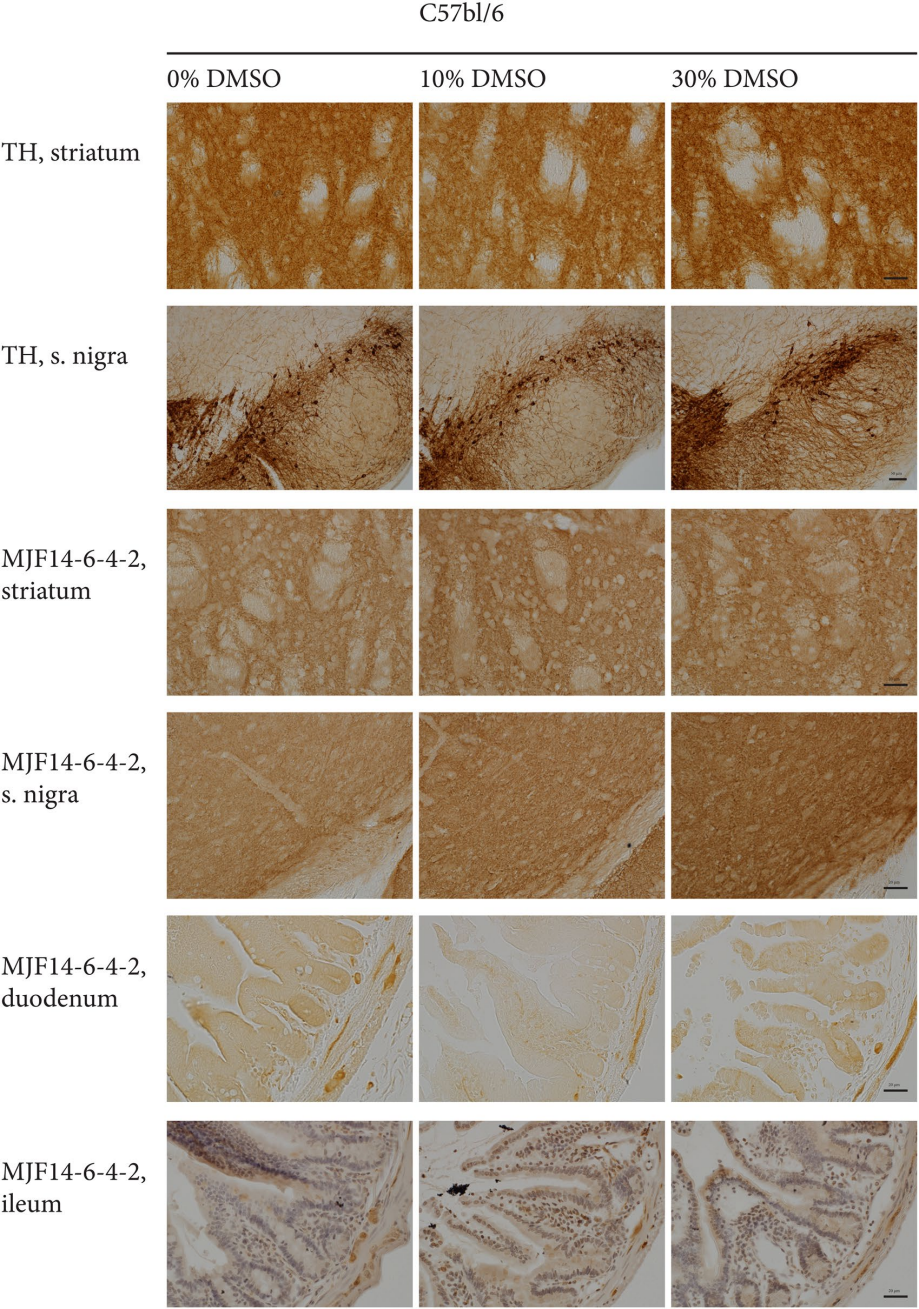
Oral treatment with DMSO does not induce α-syn aggregation in vivo. Free floating 30 um sections of brain tissue from striatum or substantia nigra or paraffin embedded sections of duodenum and ileum from wt C57BL/6 mice.Twelwe wt C57bl/6 mice were divided into three groups with four in each and treated daily with water (0% DMSO), 10% DMSO (1 g/kg bodyweight) or 30% DMSO (3 g/kg bodyweight) for 14 days prior to sacrifizing and tissue collection. Striatum or substantia nigra sections from all animals were stained with an anti-tyrosine hydroxylase antibody and with the MJFR-14–6-4–2 antibody (n = 4 for each condition). The duodenum and ileum samples from all animals were stained with the MJFR14-6–4-2 antibody. n = 4–6 sections per segment (s2, s5, s8) were analyzed for the intestinal samples and one series of brain sections spanning the entirety of the brain from OB to beginning of cerebellum (30 µm sections, 10 series = 300 um distance between the sections for each series) for each animal. Scalebar = 20 µm for striatum, duedenum and ilium and 50 µm for substantia nigra.

When analyzing the duodenum and ileum of C57bl/6 mice, specific MJFR-14-6-4-2 α-syn immunopositive profiles could be seen in all experimental groups (Fig. 4). The structures localized both to the muscular layer, in typical enteric plexi formations and in the mucosal cells, where enteroendocrine cells could be present (Fig. 4). However, no visible difference in MJFR-14-6-4-2 reactivity between the different treatments was observed (Fig. 4). In the brain, a diffuse staining pattern without any clear positive cellular structures was observed when using the MJFR-14-6-4-2 antibody (Fig. 4). This pattern was evident both in the striatum and in the substantia nigra across all treatment groups (Fig. 4). To test if the lack of MJFR-14-6-4-2 positive structures between water-, 10% DMSO-, and 30% DMSO treated C57bl/6 mice was due to the low endogenous expression level α-syn we analyzed the gastrointestinal tract and brain of DMSO-treated F28 mice overexpressing human α-syn. Yet, despite an overexpression of human α-syn, we did not detect any visible differences in MJFR-14-6-4-2 positive structures between water- and DMSO treated animals in any of the stained regions (Supplementary Fig. 6). Finally, no visible differences in the number of tyrosine hydroxylase positive cells were observed between animals, suggesting that the DMSO treatment did not induce cell death of dopaminergic neurons in the striatum or substantia nigra within the time frame of this experiment (Fig. 4 and Supplementary Fig. 6). Taken together, these data indicate that oral DMSO treatment did not have an observable effect on α-syn aggregation nor dopaminergic neuron survival in young wildtype C57bl/6 or F28 transgenic mice overexpressing human α-syn.

## Discussion

Due to DMSOs versatility as a polar, aprotic and amphiphilic molecule, it continues to serve as one of the most popular solvents in scientific research used to dissolve a variety of polar and nonpolar constituents. Yet, in line with our observations, multiple reports suggest that DMSO can induce gross changes even at low concentrations, when applied in scientific research experiments. Other researchers have found that low DMSO concentrations induce compaction of the native protein structure in hen egg-white lysozyme and myoglobin^33^, while similar low DMSO concentrations (0.5–3%) treatment of human growth hormone receptor and the phosphatase domain of PFKFB1 led to protein structure destabilization and aggregation^34^. On the other hand, opposing stabilizing and destabilizing effects of avidin and CYP142A1 were induced by DMSO, suggesting that the effect of DMSO is highly protein-dependent^35^.

DMSO treatment evidently also affects cell signalling and macromolecular structure in multiple ways. For instance, more than 2000 genes were differentially expressed upon 0.1% DMSO treatment of microtissues compared to non-treated conditions^25^. Similarly, low dose DMSO treatment (0.1–1.5%) of cells led to a comprehensive alteration of especially protein- and nucleic acid organization. Here a shift towards β-sheet structure was observed for proteins in a dose-dependent manner^24^. Based on our in vitro*-* (Fig. 1) and cellular experiments (Fig. 3), which suggest that α-syn multimerization occurs in the presence of DMSO, as well as previous reports showing that α-syn adopt β-sheet structure upon oligomerization and fibrillation^16,36^, we cannot exclude that α-syn is one of these cellular proteins adopting β-sheet structure upon DMSO treatment.

The underlying mechanism of α-syn multimerization during DMSO exposure is unknown, but DMSO can influence the solubility of specific amino acids. Glycine solubility in aqueous DMSO is low compared to water, while this decrease in solubility is less pronounced in hydrophobic amino acids such as alanine and leucine^37^. On the other hand the solubility of tryptophan increases in aqueous DMSO, suggesting that DMSO does still interact with hydrophobic amino acid residues^37^. In agreement with this, a different study described non-covalent interactions between the sulfoxide group of DMSO and aromatic side chains^38^. A possible mechanism of DMSO induced aggregation, could therefore involve disassembly of intramolecular interactions by disruption of hydrophobic clusters via its sulfoxide group, potentiating intermolecular interactions leading to nucleation and aggregation. The extent of oligomerization is uncertain from the presented results. However enhanced MJFR14-6-4-2 antigenicity on dot blot is evident after just 30 min of DMSO treatment even at low α-syn concentrations (10 µg/ml, 0.69 µM) (Fig. 1), and in cells at longer DMSO exposure times (Fig. 3). Furthermore, we do observe proposed α-syn dimerization by BiFC in cells (Fig. 3). It has to be stated that the MJFR14-6-4-2 antibody has been shown to also detect monomeric α-syn^39^, however when applied in low concentrations it shows a strong preference for aggregates (Supplementary Fig. 2 and^40^). Our in vivo data on the other hand do not suggest that oral DMSO treatment in mice can induce gross changes to enteric α-syn or α-syn in the brain within 14 days (Fig. 4 and Supplementary Fig. 6). However, we do not know how much of the orally administered DMSO that actually reaches the gut, how long time the cells are exposed, and at what concentration. Yet, given the high concentrations of DMSO used in our experimental setup in vivo, we do not expect the low DMSO concentrations usually applied in animal research to interfere with, or perturb, the structure of α-syn in vivo, and therefore see no reason to substitute DMSO with another solvent when working with animals.

The DMSO concentrations we applied in our cell culture experiments on the other hand (0.1–0.75%), are easily within the range normally used to treat cells, e.g. when DMSO is used as a co-solvent for drug testing. Although a final concentration of 0.1–0.75% does not appear particularly high, the molar concentration of 0.75% DMSO, as calculated as a volume percentage in this work, translates into 106 mM. This means that DMSO at 0.75%, is the third most prevalent molecule in the culture media, but already at 0.1% (14 mM) DMSO is relatively abundant. Concentrations that high of any molecule, will undoubtedly affect the cells, and a number of these cellular effects introduced by DMSO could induce aggregation indirectly. Nevertheless, combined with the direct induction of aggregation, which we detect in vitro, a direct interaction between DMSO and α-syn appears the likely causative effect behind the observed cellular α-syn aggregation. Interestingly, we did not observe increased phospho-Ser129 levels of α-syn upon DMSO treatment. This could be due to the nature of the aggregation-like cellular α-syn structure that might serve as poor kinase substrates compared to physiological α-syn aggregates.

Our data clearly suggest that precautions should be taken when addressing questions regarding the native structure of α-syn in the presence of DMSO. One experimental setting where DMSO hypothetically could play an unfortunate and misleading role, is within the field of α-syn tetramerization^4,41^. DMSO has routinely been used to solubilize cross-linkers, and in experiments used to verify the existence of α-syn tetramers in cells, DMSO was present in a 0.2% final concentration^4,41^. Our experiments suggest that DMSO in this concentration is sufficient to induce vast multimerization of α-syn in cells (Fig. 3). Based on these findings we propose that the existence of α-syn tetramers ought to be verified using water-soluble cross-linkers.

DMSO is also frequently used as a co-solvent in drug effect screens on α-syn aggregation^42,43^, and testing the effect of various drugs on α-syn aggregation is often accompanied by a buffer-control with DMSO. However, our results on DMSO induced aggregation, suggest that the assayed effects would still be on a perturbed conformational state of α-syn. Careful consideration should therefore be made when looking at α-syn aggregation, and this work indicates that biochemical experiments on α-syn might be better suited, using a different solvent or water soluble reagents. At the very least, one should consider preparing high concentration stock solutions of the used compounds, in order to dilute DMSO to the highest possible extent when conducting α-syn-related research in vitro and in cells.

Contrary to our in vitro*-* and cell-based assays, our in vivo experiments did not indicate any signs of DMSO-induced α-syn aggregation in wt C57BL/6- or transgenic F28 mice. Our mice received a daily oral dose corresponding to 1% of their bodyweight (e.g. 0.3 mL for a 30 g mouse) containing pure water, 10% DMSO or 30% DMSO. The overall fluid content in the gastrointestinal tract in adult mice has previously been determined to approximately 1 mL^44^. In our case, an additional 0.3 mL will result in an average DMSO concentration of ≈ 2.3% (in the 10% DMSO treated animals) or ≈ 6.9% (in the 30% DMSO treated animals) throughout the gastrointestinal tract. Yet, locally the concentration might be even higher. Nevertheless, we did not observe any post-mortem α-syn abnormalities in these areas (Fig. 4 and Supplementary Fig. 6). This could be due to low expression levels of α-syn in the cells lining the gastrointestinal tract or the transient daily administration.

If we look at the plasma DMSO levels it naturally looks a lot different. DMSO is readily dissolved in water, and easily absorbed in the body. Yet it is also excreted, and in monkeys DMSO’s half-life in blood was calculated to 38 h^45^. Moreover, a continued daily oral administration of 3 g DMSO / kg bodyweight, identical to the highest treatment in our experimental setup, resulted in a steady state DMSO serum concentration of 0.9 mg/ml (translated into 0.09% DMSO or 12.6 mM)^45^. At these concentrations we begin to see differences in α-syn aggregation in our cell assays (Fig. 3), however, it was not sufficient to induce α-syn abnormalities, nor cytotoxicity in different brain areas (Fig. 4 and Supplementary Fig. 6). Again, the levels of α-syn might be higher in our cell-lines compared to in vivo conditions, but another explanation could also be that DMSO is removed more rapidly in mice compared to monkeys due to differences in metabolism. The latter claim is based on previous studies, showing that 60% of oral ingested DMSO in primates are excreted in the urine^45^, and the finding that 10 different test compounds on average were excreted 13 times faster (mL/min/kg) in mice compared to humans^46^. Based on our data, oral administration of DMSO in mice should not be a cause concern in relation to studying the overall structure of α-syn.

## Materials and methods

### Antibodies

Primary antibodies: α-syn monoclonal mouse-anti-α-syn (SYN-1, BD Biosciences, catalog no. 610787), polyclonal rabbit-anti-α-syn (ASY1^47^), rabbit-anti-α-syn (phospho S129) (abcam, catalog no. ab51253), rabbit-anti-α-syn (phospho S129) (Cell Signaling, D1R1R, mAb #23,706), MJFR-14-6-4-2 (MJF14) (abcam, catalog no. 209538, rabbit monoclonal antibody, generated against full length α-syn protein filament), anti-α-tubulin (T9026 Sigma-Aldrich Co.) and anti-Tyrosine Hydroxylase (GeneTex, GTX113016). Secondary antibodies: horseradish peroxidase (HRP)-conjugated polyclonal rabbit-anti-mouse (Dako, P0260), HRP-conjugated polyclonal swine-anti-rabbit (Dako, P0217).

### Production and purification of α-syn

Human recombinant α-syn and β-syn were purified from transformed BL21(DE3) competent cells as previously described^48,49^. Briefly, protein purification involved dialysis of proteins against 20 mM Tris pH 6.5 overnight, followed by ion-exchange chromatography on a Poros HQ50 column (Thermo Fisher Scientific) with a 0–2 M NaCl gradient. Next, a reverse phase-high pressure liquid chromatography purification step was performed on a Jupiter C18 column (Phenomenex, Torrance, CA) in 0.1% trifluoroacetic acid with an 0–90% acetonitrile gradient. Isolated proteins were dialysed in PBS pH 7.4 overnight followed by an additional dialysis step in 20 mM ammonium bicarbonate overnight. Protein concentration was determined by bicinchoninic acid (BCA) protein concentration assay (Pierce). The proteins were subsequently aliquoted, lyophilized, and stored at − 20 °C until use.

### Dynamic light scattering (DLS)

For size determination using DLS, recombinant α-syn or carbonic anhydrase were spin-filtered through a 100 kDa filter (Amicon Ultra-0.5 Centrifugal Filter Unit), incubated for 1 h at a concentration of 0.5 mg/mL (equivalent to 35 µM of α-syn or 17 µM of carbonic anhydrase) in phosphate-buffered saline (PBS, pH 7.4) alone or in combination with 2%, 5% or 10% DMSO at room temperature (RT), and placed in a disposable plastic cuvette. Particle size within the samples was analyzed as previously described^16^ using a Wyatt Technology DynaPro NanoStar. Laser strength was set to 100% and scatter angle fixed at 90°. Total sample measurement was assembled via ten consecutive 5 s measurements. Data was analyzed with Dynamics V7.5.0.17 with solvent signal subtracted from each sample.

### Immunoblotting

Dot blotting was performed on 100 ng of protein blotted on nitrocellulose (Hydrobond-C Extra, GE Healthcare). Prior to loading, α-syn was incubated for 30 min at a concentration of 10 µg/mL (0.69 µM) in PBS alone or in combination with 0,1%, 0,5%, 1%, 2%, 5% and 10% of DMSO at room temperature (RT). All membranes were blocked in non-fat milk (Tris-buffered saline (TBS) with 5% non-fat milk, 0.05% Tween 20 and 0.02% sodium azide) for 30 min at RT followed by incubation with primary antibodies overnight. Membranes were washed in TBS-T (TBS with 0.1% Tween) and incubated with secondary antibodies for 1.5 h. Membranes were washed, developed using ECL reagent (Pierce™, Thermo Scientific), and subsequently imaged on a LAS-3000 imaging system (Fuji). Blot quantification was done using ImageJ.

### Oligomer preparation

Oligomers were prepared as previously described^39^. Briefly, 12 mg/mL α-syn monomer was dissolved in PBS and incubated at 37 °C at 900 rpm for 5 h. After incubation, the sample was centrifuged at 12000×*g* for 5 min and the supernatant loaded onto a Superdex™ 200 10/300 GL column (GE Healthcare). The column was eluted with PBS at a flow rate of 0.5 mL/minute. Oligomers were collected between 18–22 min and monomers after 38–43 min. These oligomers were then either used directly or cross-linked with formaldehyde using an previously established protocol^16^.

### Fibrillation

Lyophilized recombinant α-syn or β-syn were re-suspended in sterile PBS (Gibco) to a final concentration of 0.5 mg/mL (35 µM) and placed in Eppendorf Thermotop shaker at 37 °C, 1050 rpm for 17 days. For ThT measurements 10 µl of samples was added to 100 μl of 40 μM ThT (final concentration) dissolved in measure buffer (90 mM glycine, pH 8,6) and incubated for 5 min at RT before measuring ThT signal on an EnSpire 2300 Multilabel Plate Reader (PerkinElmer Life Sciences) (excitation at 450 nm and emission at 486 nm). Values were normalized to day 0 measurements for each sample and fitted as a sigmoidal growth curve using GraphPad Prism 8.3.0 software. For sedimentation assay, the sample was centrifuged at 25.000×*g* for 30 min, at 20 °C. Supernatant was collected and considered as soluble fraction, while insoluble fibrils in the pellet fraction were washed and re-suspended in PBS, in the same volume as the isolated supernatant. Similar volumes of supernatant and pellet fractions were resolved then diluted 1:1 in Special SDS-loading buffer (100 mM Tris pH 6.8, 8% SDS, 24% glycerol, 0.02% bromophenol blue, 25 µM 1,4-Dithioerythritol), boiled for 5 min, thoroughly vortexed and left at RT for 30 min. The samples were then boiled another 5 min to ensure complete dissociation of fibrils. Subsequently the samples were subjected to SDS-PAGE and stained at RT, using Coomasie Brilliant Blue. The gel was destained ON at RT and photographed on Fuji LAS-3000 Intelligent Dark Box (Fujifilm, Japan).

### Transmission electron microscopy

Transmission electron microscopy (TEM) was performed as described previously^50^ with minor modifications. In brief, a 5 µl sample was applied to a 400-mesh carbon-coated, glow-discharged Ni grids for 30 s. The grids were stained with 2% uranyl formate and dried. Samples were viewed on a Tecnai G2 Spirit (FEI company) operating at 120 kV, and images were taken using a TemCam F416 camera (TVIPS) at a magnification of 26000x.

### Proteinase K digestion

To assay the conformational differences between the PFFs generated, the fibrils were digested with proteinase K (PK). Prior to digestions, PFFs were centrifuged at 25.000×*g*, at 20 °C re-suspended in PBS to remove DMSO. 20 μL of 2 mg/mL (138 µM) PFF (40 μg) was diluted 1:1 in digest buffer in an Eppendorf tube. PK (Sigma- Aldrich) was diluted in digest buffer and added to the PFF samples at final concentrations of 50, 10 and 2 μg/mL. A control PFF sample was run as control without PK. The samples were incubated at 37 °C with slow agitation for 30 min. Afterwards the reaction was stopped using 5 μL of Pefabloc (Sigma-Aldrich) and left at RT for 10 min. Samples were then diluted 1:1 in Special SDS-loading buffer , boiled for 5 min, thoroughly vortexed and left at RT for 30 min. The samples were then boiled another 5 min to ensure total dissociation of fibrils. Samples were applied on a 10 well Novex™ 16% Tricine Gel (Invitrogen™) and stained using Coomassie Brilliant Blue.

### Cell cultures

All cell lines were maintained as previously described^51^. In brief, OLN-AS7 cells^52^ expressing human α-syn were made from an immortalized oligodendroglia cell line (OLN-93^53^) by stable transfection with a pcDNA3.1 zeo( −)α-syn vector, and maintained with 100 μg/mL zeocin (Invitrogen). These cells were cultured at 37 °C under 5% CO2 and grown in DMEM (Lonza) supplemented with 10% fetal calf serum, 50 U/mL penicillin, and 50 μg/mL streptomycin. For experiments, cells were seeded without zeocin 24 h before treatment.

HEK293T cells (kindly provided by Professor Jacob Giehm Mikkelsen, Department of Biomedicine, Aarhus University) were cultured as described for OLN-93, but without zeocin treatment.

The SH-SY5Y cell line, is a sub-cloned variant of the human neuroblastoma SK-N-SH cell line. We used a variant (SH-SY5Y ASYN) that stably overexpressed wt human α-syn via a Tet-Off-system^22^, kindly provided by Professor, Dr. Leonidas Stefanis, Division of Basic Neurosciences, Biomedical Research Foundation of the Academy of Athens, Athens, Greece. Here, α-syn expression is suppressed by doxycycline (dox). Mitosis was inhibited by administration of 10 μM retinoic acid (RA) final concentration. SH-SY5Y ASYN cells were cultured at 37 °C under 5% CO2 in RPMI 1640 medium with l-Glutamine (Lonza) supplemented with 15% FCS, 50 U/mL penicillin and 50 μg/mL streptomycin. Cells were maintained in 50 μg/mL hygromycin B (Invitrogen, Life Technologies), 200 μg/mL geneticin (VWR), 1 μg/mL dox (VWR), and 5 μg/mL Plasmocin. For experiments, cells were seeded without hygromycin B, geneticin, plasmocin.

### Neuronal differentiation of SH-SY5Y cells and cell viability assay

For this assay, neuroblastoma SH-SY5Y ASYN cells, with inducible α-syn expression controlled by the Tet-Off system, were differentiated into a neuronal lineage. For this purpose we used a previously published protocol^22^, albeit using a lower serum concentration of 2.5% instead of 10%, to yield a more pronounced differentiation and block of mitosis. The experiment was performed as follows: at day 0 5000 cultured SH-SY5Y ASYN cells were plated to 96-well plates in RPMI 1640 medium (Lonza, Cat.No BE12-702F) without doxycycline and supplemented with 5% heat-inactivated fetal bovine serum (hiFBS) (Biowest, Cat.No S1810), 100 U/ml penicillin and 100 ug/mL streptomycin (Merck-Millipore, Cat.No A2213), and were kept at 37 °C under 5% CO2. From day 1, 20 μM all-trans retinoic acid (RA) (Sigma, Cat.No R2625) was also supplemented to the differentiating medium (RPMI 1640 medium with 2.5% hiFBS). On day 2 the cells were treated with PFF (28 μg/mL) which was removed on day 4. From day 2 the medium was refreshed in every second day. On day 8 the viability of the cells was evaluated by metabolic activity in an MTT assay (Life Technologies) according to the manufacturer protocol. In brief, Thiazolyl Blue Tetrazolium Bromide (Sigma, Cat.No M5655) was administered to the cells and incubated at 37 °C for 7 h, then the cells were lysed overnight, and the absorbance were detected at 570 and 630 nm in every well using a PerkinElmer EnSpire 2300 Multilabel plate reader.

### Seeding of cellular aggregation

For immunocytochemistry OLN-AS7 cells were seeded onto poly-l-lysine-coated coverslips and 24 h post-seeding exposed to 14 µg/mL (1 µM) α-syn PFFs made in the presence or absence of 2% DMSO or left untreated. Before addition to cells, the PFFs were sonicated (20 min, using a Branson Ultrasonics™ Sonifier S-250A, duty cycle 30 and output control 3, in a water bath continuously renewed by cold running water to prevent heating.) to produce a uniform population of elongation-prone seeds. The α-syn PFFs were made from either monomeric wt α-syn or monomeric S129A substituted α-syn which cannot be phosphorylated on the S129 residue. After 24 h PFF treatment, the cells were washed and incubated for another 24 h before washing in PBS and fixation in 4% PFA for 10 min. Finally the coverslips were washed in PBS and incubated briefly in 15 mg/mL NH_4_Cl in PBS. Coverslips were then blocked and incubated with primary and secondary antibodies in the blocking buffer (PBS, 3% bovine serum albumin (BSA), 0.1% saponin), with washings in between in PBS with 0.1% saponin and finally double-distilled H_2_O, before mounting with Mounting Medium (DAKO, S3023) on glass slides and left in dark O/N at 4 °C. Images were taken with X20 and X63 objectives on a Zeiss ObserverZ1 microscope, and pictures were quantified with ImageJ. MJFR14-6-4-2 and anti-phospho-Ser129 α-syn signals were quantified by taking the area above a set threshold based on the control sample, and normalizing it to the number of nuclei based on DAPI staining. For each experiments ~ 10 images quantified for each of three independent experiments.

### DMSO induced α-syn aggregation in neuron-like cell model

Neuroblastoma SH-SY5Y ASYN cells with inducible α-syn expression controlled by the Tet-Off system were seeded on poly-L-lysine coated coverslips and differentiated with 10 μM retinoic acid. Retinoic acid was included in the media for the duration of the experiment. Induction of α-syn expression were started 24 h after seeding, and the cells were simultaneously treated with DMSO for 7 days, with media renewal every second day. The cells were then fixed, blocked and then probed with anti-tubulin (1:1000) and MJFR14-6-4-2 (1:25,000). Fluorescently labeled secondary antibodies anti-mouse (1:1000) and anti-rabbit (1:1000) and DAPI were subsequently added. Images were taken with X20 and X40 objectives on a Zeiss ObserverZ1 microscope, and pictures were quantified with ImageJ. MJFR14-6-4-2 and anti-phospho-Ser129 α-syn signal were quantified by taking the area above a set threshold based on the negative control, and normalizing it to the area of signal from α-Tubulin. For MJFR14-6-4-2, four independent experiments were conducted with ~ 10 images quantified for each experiment. For anti-phospho-S129 α-syn, three independent experiments were conducted with ~ 8 images quantified for each experiment.

### Bimolecular fluorescence complementation visualization of α-syn oligomers

Venus-YFP vectors described in^26,27^ for BiFC visualization of α-syn oligomers were developed and kindly provided by Professor Pamela J McLean (Mayo Clinic, Jacksonville, USA). In short, fluorescent venus YFP was used generating the fluorescent complementation pairs V1S (N-terminal half of Venus YFP fused to the N-terminus of α-syn) and SV2 (C-terminal half of Venus YFP fused to the C-terminus of α-syn). For experiments, 500.000 HEK cells were seeded into 6-well plates and transfected with V1S or SV2 alone or in combination using Lipofectamine 3000 (Thermo Fisher Scientific) transfection reagent according to the manufacturer's protocol. One day post transfection 25.000 cells were re-seeded into each well of a Poly-L-lysine-coated 96-well plate. DMSO was added to the media in described concentrations four hours post re-seeding. Cells were incubated for an additional 20 h before analysis on an EnSpire 2300 Multilabel Plate Reader (PerkinElmer Life Sciences) (excitation at 513 nm and emission at 528 nm).

### Animal experiments

All animal experiments were conducted in accordance with ethical permits approved by the local Animal Welfare and Ethics Committee Malmö/Lund (ethical permit numbers M73/16 and the amendment5.8.18–03,524/2020). Furthermore, all animal experiments were performed in accordance with relevant guidelines and regulations and reporting in the manuscript follows the recommendations in the ARRIVE guidelines. Twelve wildtype C57bl/6 mice and 12 transgenic F28 mice expressing human full-length wildtype α-syn under the mouse’s endogenous α-syn promoter were kept in a 12-h light/12-h dark cycle with access to food and water ad-libitum. At 4 months of age, the mice were given a single dose of PBS, 10% DMSO (1 g/kg) or 30% DMSO (3 g/kg) by oral gavage daily for 2 weeks. The animals were weighed daily and closely monitored. At the end of the 2 weeks, the mice were injected intraperitoneally with 0.6 mg/g sodium pentobarbital. After loss of righting reflexes, the mice were transcardially perfused with 0.9% saline. Brains and gastrointestinal tracts were collected and either snap frozen in liquid nitrogen or post-fixed in 4% PFA pH 7.4 for 48 h. Tissues were then either cryopreserved in 30% sucrose or prepared for paraffin embedding in 70% ethanol and stored at 4C.

### Immunohistochemistry

Brain samples were cut in 30 µm coronal sections on a freezing microtome and stored in Walter’s antifreeze solution. Sections were washed in PBS and endogenous peroxidase quenched with 3% hydrogen peroxide. Sections were pre-incubated in a solution with normal serum, BSA and Triton-X100. Sections were incubated with primary antibody solutions (MJFR14-6-4-2 antibody (1:5000) or (1:300,000) GTX113016) and secondary antibody solution (1:500 BA-1000), amplified with ABC solution (VEPK-6100), and developed with DAB (SK-4100). Sections were mounted onto gelatin-coated glass-slides, dehydrated, cleared and coverslipped with pertex.

Intestinal segments from the duodenum and jejunum were paraffin embedded and sectioned at 6 um thickness. Samples were deparaffinized before antigen retrieval for 10 min in 80% formic acid at room temperature and 40 min in 1 M citrate buffer pH 8.5 at 95 °C. Immunohistochemistry was then performed as described with MJFR14-6-4-2 antibody (1:10,000) at 4 °C overnight. After developing with DAB, samples were counterstained with HTX before dehydration, clearing and coverslipping. All stained tissues were imaged with Olympus BX53.

### Statistical analysis

All statistical analyses were performed using GraphPad Prism 7 (Graph Pad software). Comparison of groups was done by one-way ANOVA followed by Tukey’s (when comparing all groups of treatment) or Dunnett’s (when comparing every groups of treatment to a control treatment) multiple comparison test. When comparing just two groups, student t-test was applied.

## Supplementary Information

Supplementary Information 1.

Supplementary Information 2.

Supplementary Information 3.

Supplementary Information 4.

Supplementary Information 5.

Supplementary Information 6.

Supplementary Information 7.
