# Supplementary figures and images for: Low dose DMSO treatment induces oligomerization and accelerates aggregation of α-synuclein
Source: Sci Rep. 2022 Mar 8;12:3737. doi: 10.1038/s41598-022-07706-2 (PMC8904838; doi:10.1038/s41598-022-07706-2)

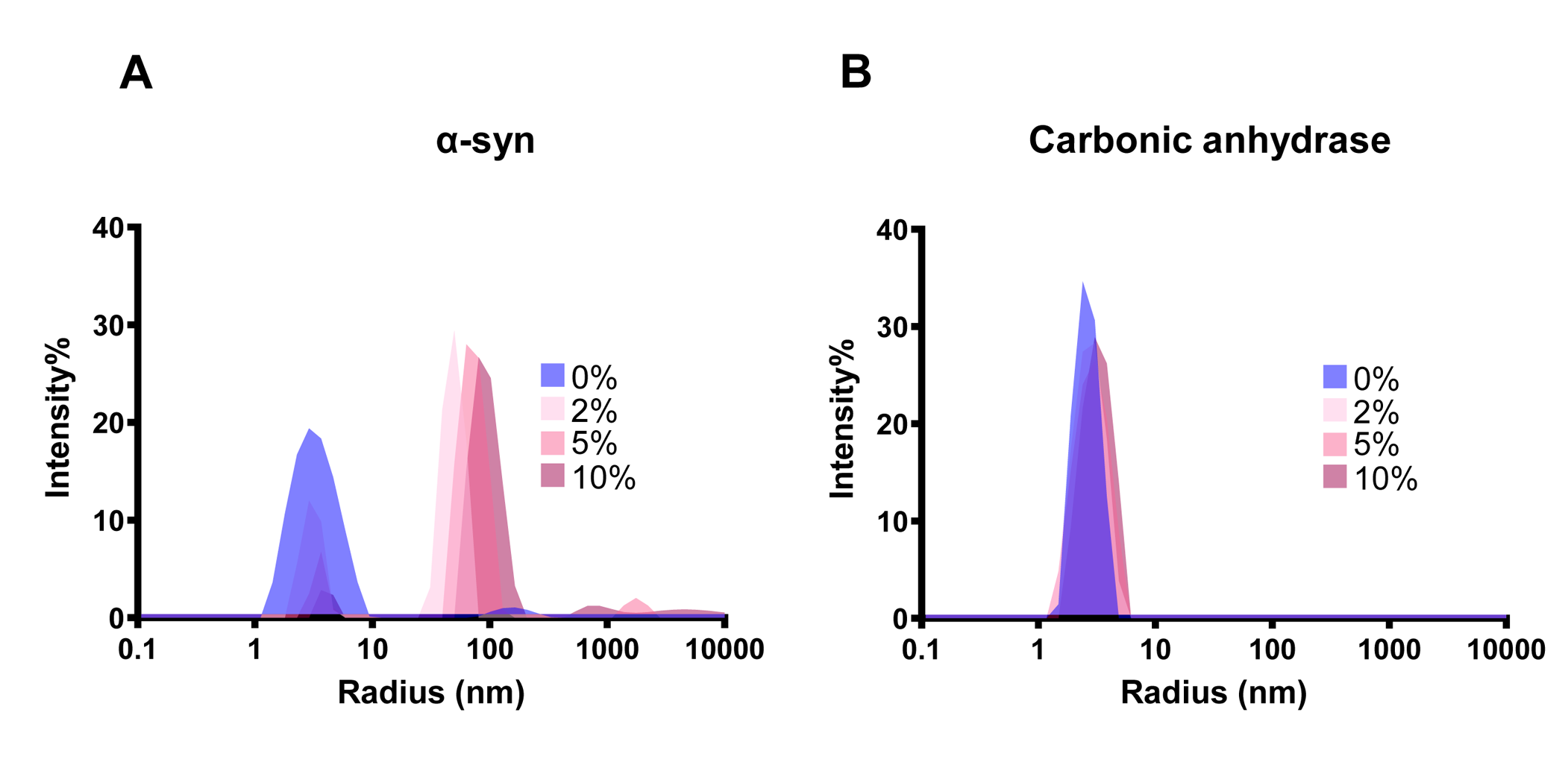

Supplement: Supplementary file 1 — Supplementary Information 1. [file 41598_2022_7706_MOESM1_ESM.tif]

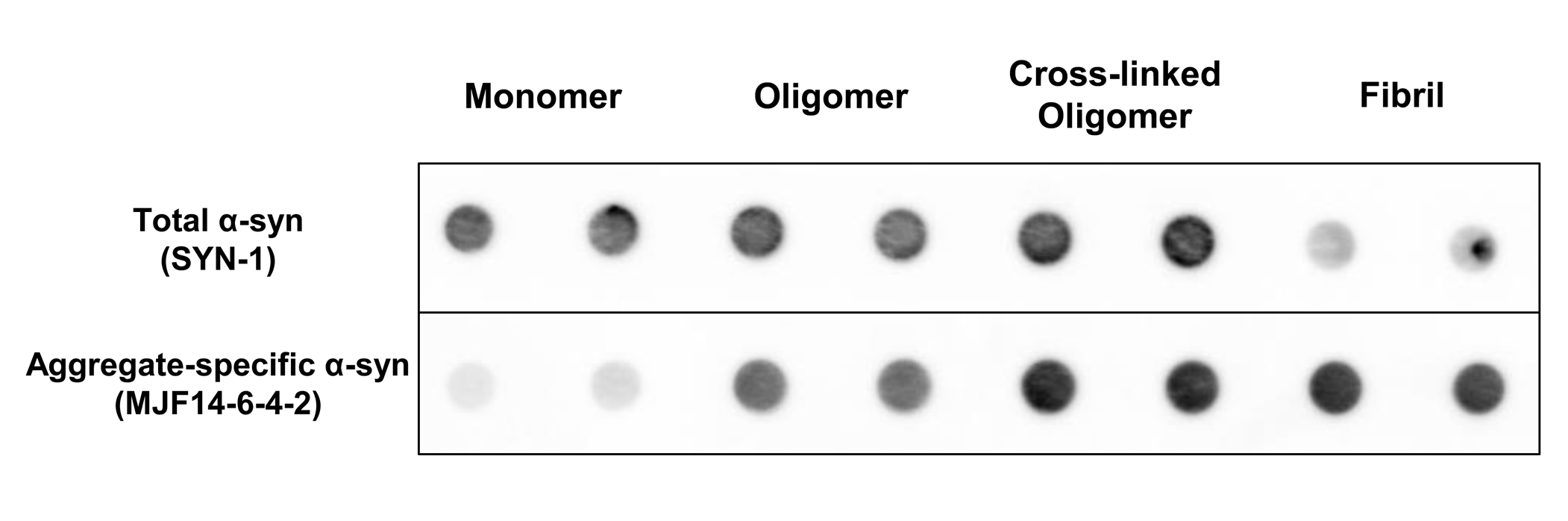

Supplement: Supplementary file 2 — Supplementary Information 2. [file 41598_2022_7706_MOESM2_ESM.tif]

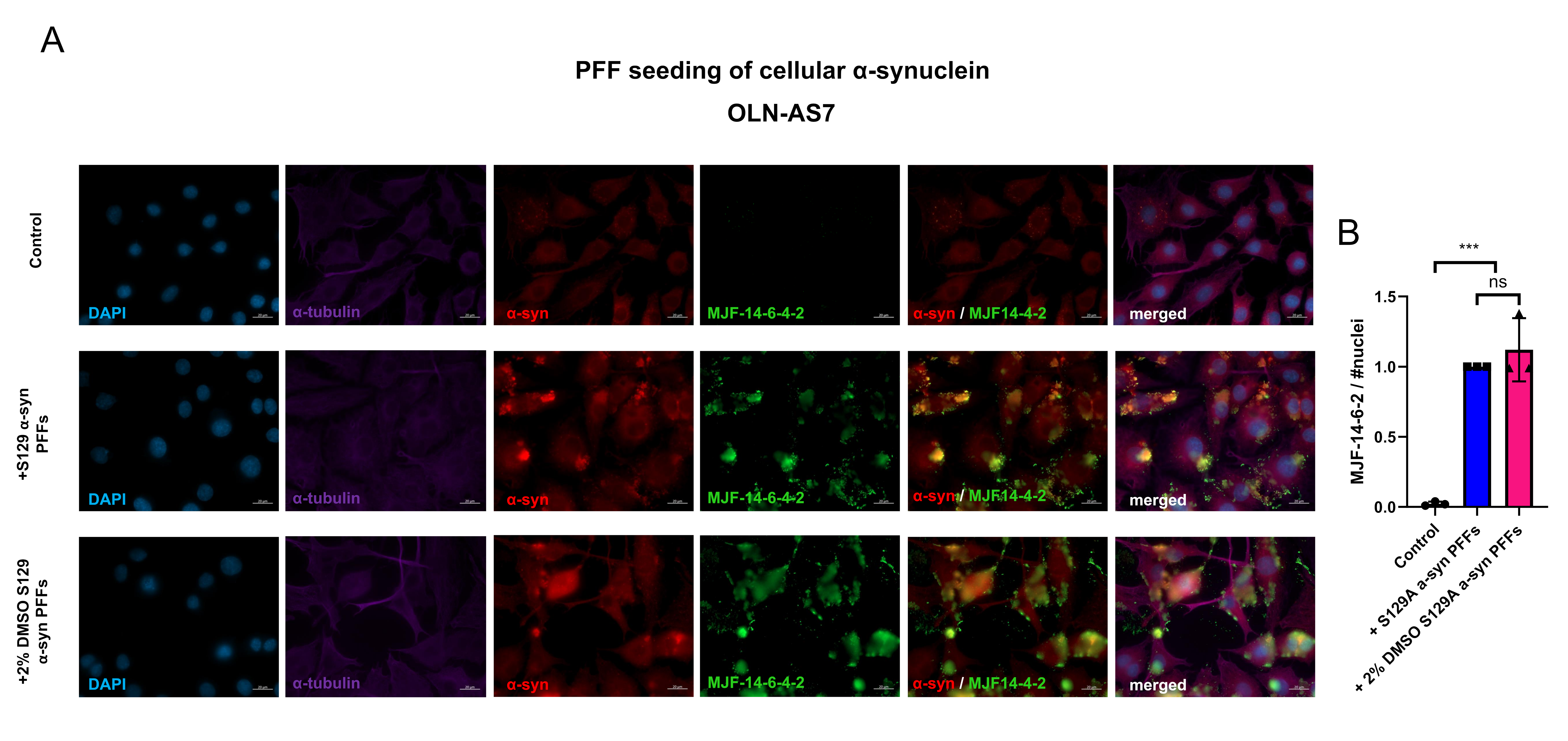

Supplement: Supplementary file 3 — Supplementary Information 3. [file 41598_2022_7706_MOESM3_ESM.tif]

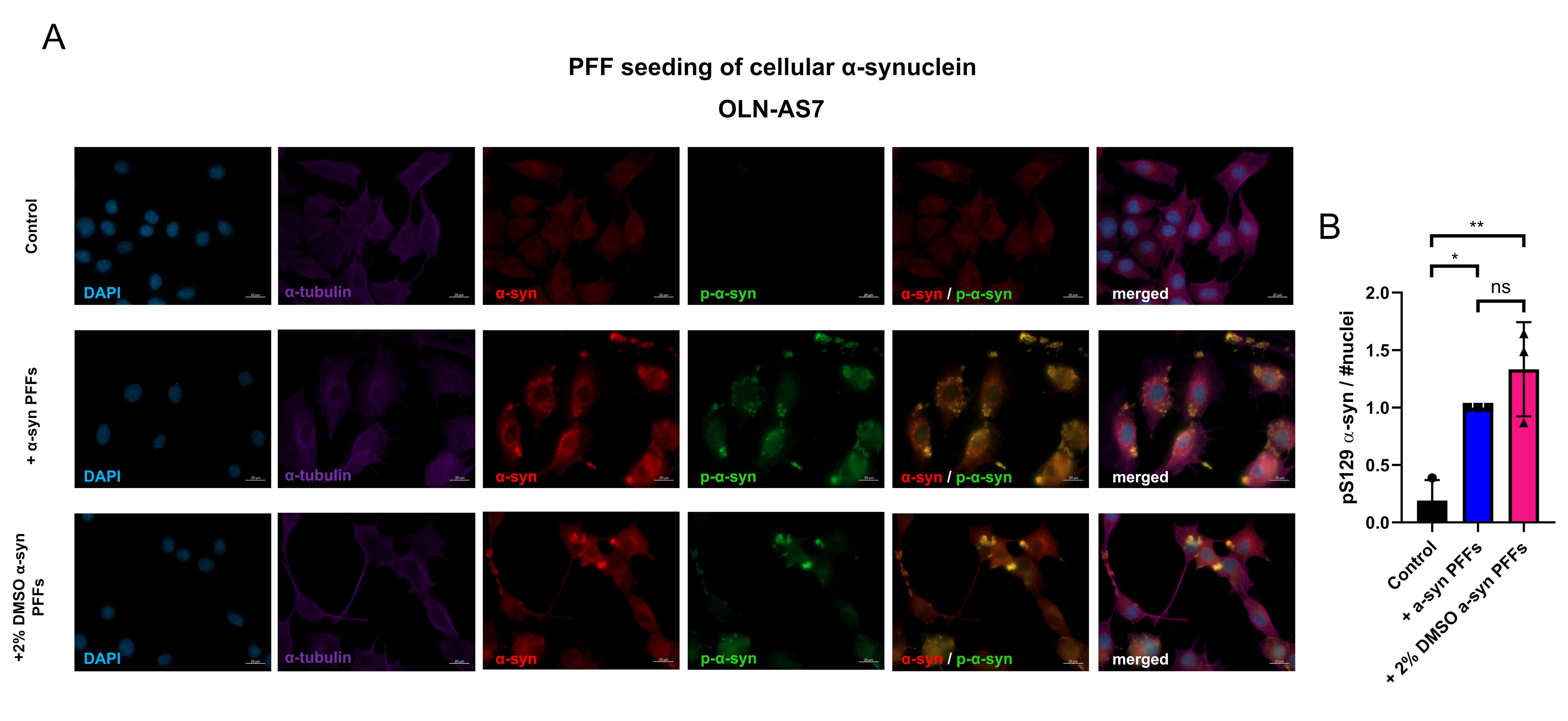

Supplement: Supplementary file 4 — Supplementary Information 4. [file 41598_2022_7706_MOESM4_ESM.tif]

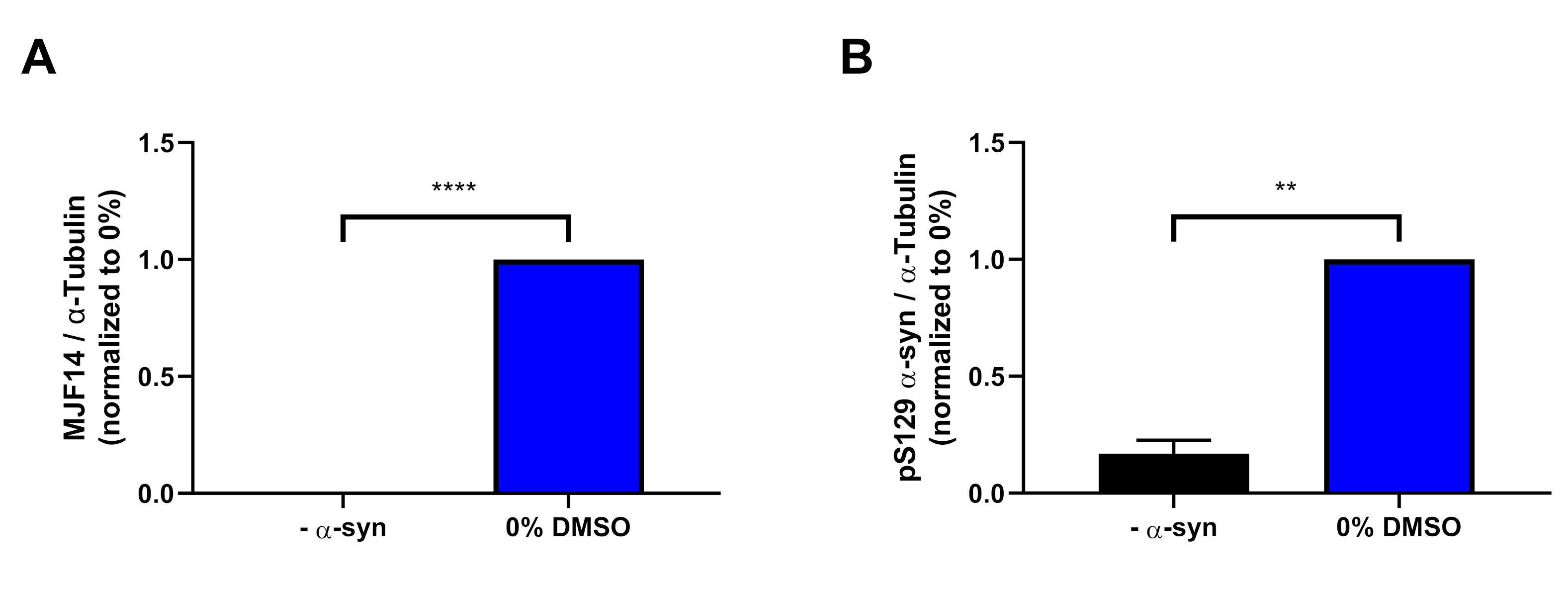

Supplement: Supplementary file 5 — Supplementary Information 5. [file 41598_2022_7706_MOESM5_ESM.tif]

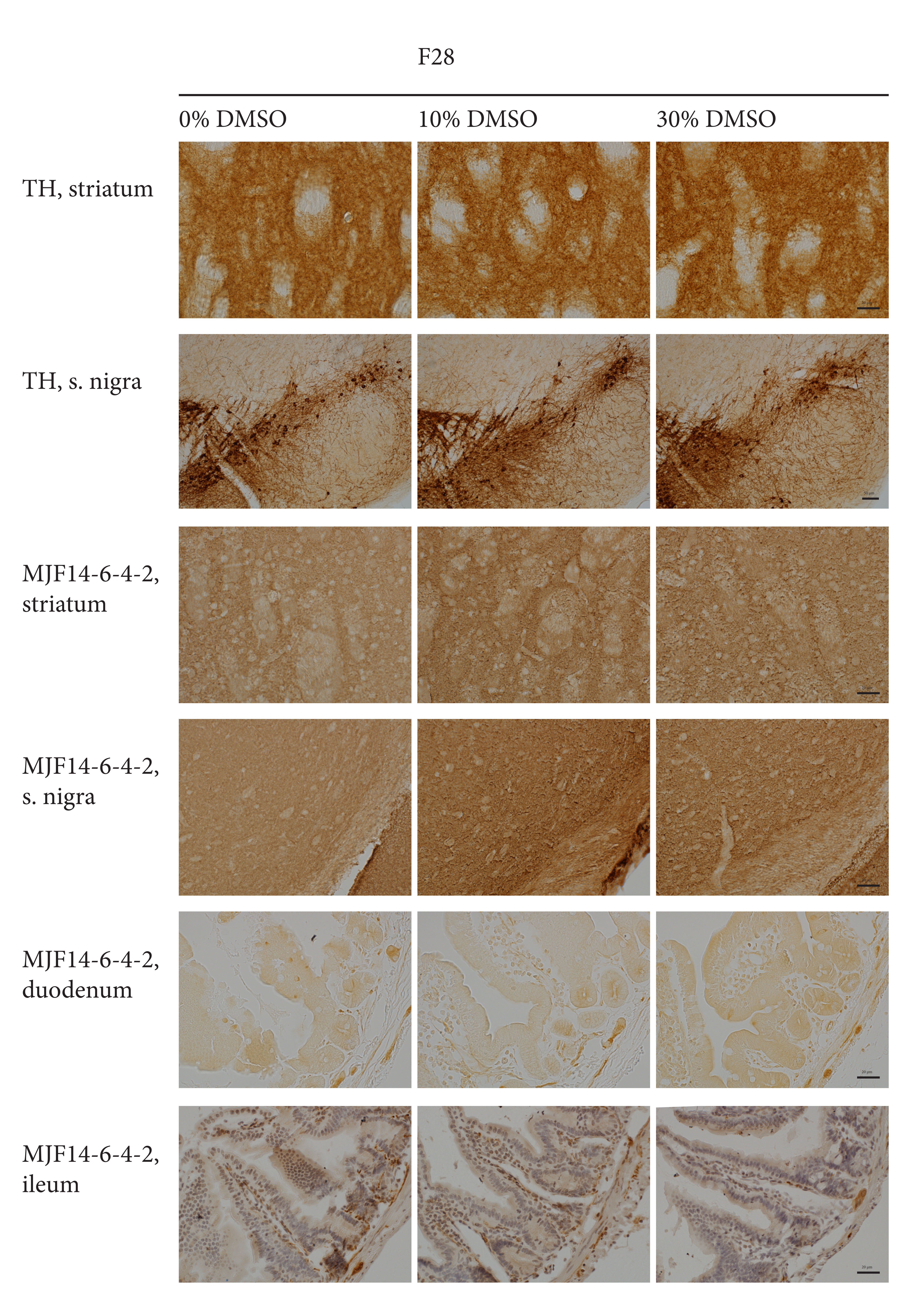

Supplement: Supplementary file 6 — Supplementary Information 6. [file 41598_2022_7706_MOESM6_ESM.tif]
