# Supplementary material for: Low dose DMSO treatment induces oligomerization and accelerates aggregation of α-synuclein
Source: Sci Rep. 2022 Mar 8;12:3737. doi: 10.1038/s41598-022-07706-2 (PMC8904838; doi:10.1038/s41598-022-07706-2)
Supplement: Supplementary file 7 — Supplementary Information 7. [file 41598_2022_7706_MOESM7_ESM.pdf]

Supplementary information

Low dose DMSO treatment induces oligomerization and accelerates aggregation of α-synuclein

Authors

*Lasse Reimer[a,b], Caroline Haikal[c], Hjalte Gram[a,b], Vasileios Theologidis[a,b], Gergo Kovacs[a,b], Harm Ruesink[a,b], Andreas Baun[a,b], Janni Nielsen[d], Daniel Erik Otzen[d], , Jia-Yi Li[c,e] and Poul Henning Jensen[a,b]

[a]Danish Research Institute of Translational Neuroscience - DANDRITE, Aarhus University, Denmark.
[b]Department of Biomedicine, Aarhus University, Denmark.
[c]Neural Plasticity and Repair Unit, Wallenberg Neuroscience Center, Department of Experimental Medical Science, Lund University, Lund, Sweden.
[d]Interdisciplinary Nanoscience Center - iNANO, Aarhus University, Denmark.
[e]Institute of Health Sciences, China Medical University, 110112 Shenyang, P. R. China

* Corresponding author

In vitro aggregation
(sedimentation of α-syn + DMSO)

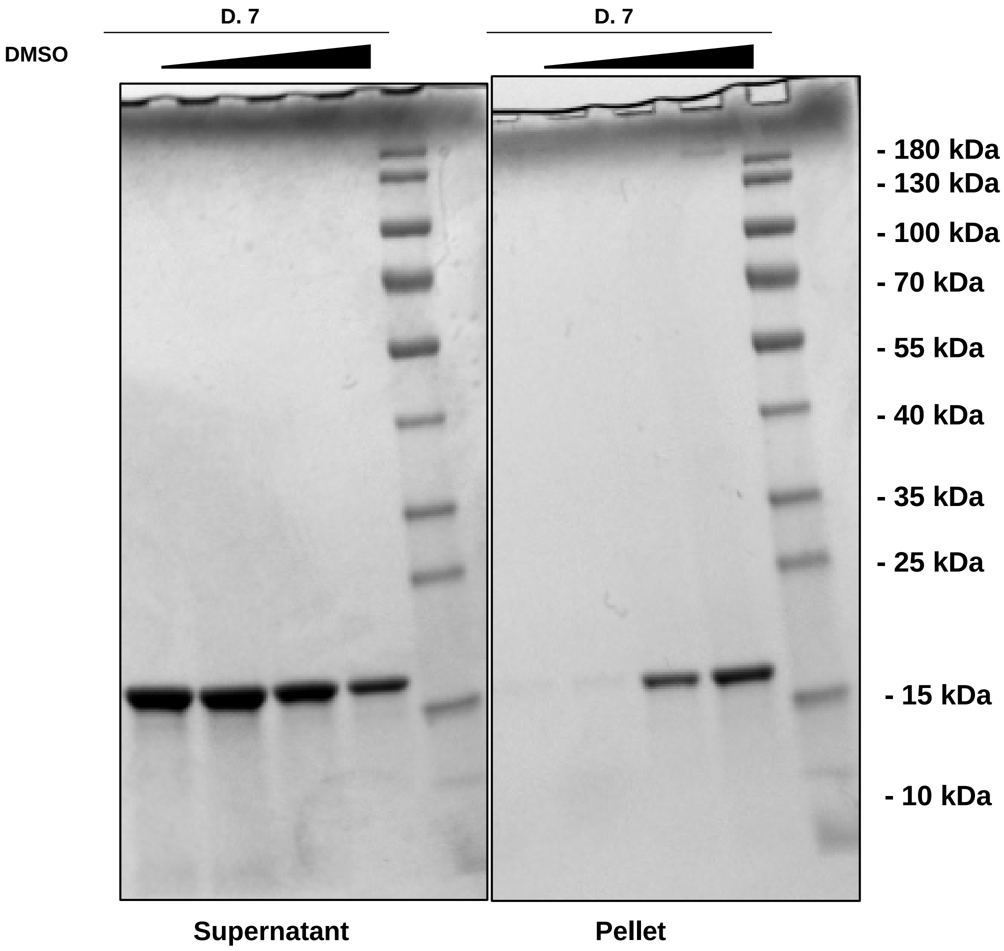

Supplementary Information 1)
Full-length SDS-PAGE gels of data presented in original figure 1F.

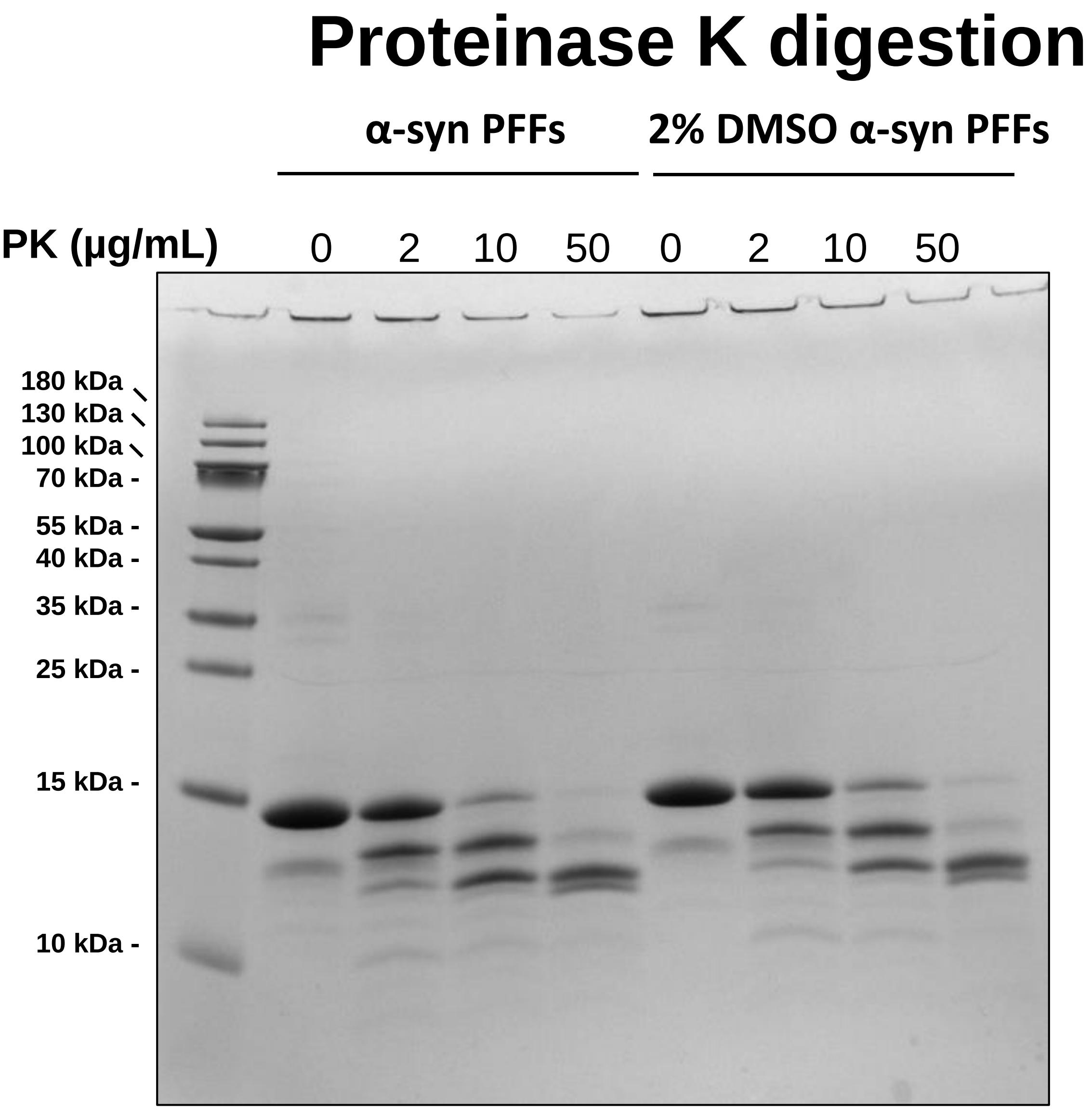

Supplementary Information 2)
Full-length SDS-PAGE gels of data presented in original figure 2B. Please note that α-syn migrates sligtly different relative to the 15kDa from the Prestained Protein Ladder on this 16% Tricine Gel compared to regular SDS-PAGE gels.

Supplementary figure legends:

Supplementary Figure 1

A and B) *Dynamic light scattering analysis of α-syn (0.5 mg/mL, 35 µM) and carbonic anhydrase (0.5 mg/mL, 17 µM) incubated for 1 h with increasing concentrations of DMSO (0%, 2%, 5% and 10%). The figure demonstrates an intensity% distribution based on the scattering intensity, with log scaled hydrodynamic radius depicted on the X-axis. Representative figure of three independent replicates (n = 3).*

Supplementary Figure 2

Dot blot of 100 ng of recombinant α-syn protein either as monomer, oligomer, cross-linked oligomer of fibrils. α-syn was visualized using total α-syn (SYN-1) and MJFR-14-6-4-2 antibodies. Representative figure of three independent replicates (n = 3).

Supplementary Figure 3

A) *Human α-syn expressing OLN-AS7 cells were exposed to PBS (Control) or 14 µg/mL (1 µM) sonicated S129A α-syn PFFs prepared in PBS alone (S129A α-syn PFFs) or in the presence of 2% DMSO (2% DMSO S129A α-syn PFFs). After 24h of PFF treatment, the cells were washed to remove excess PFF and subsequently incubated for another 24h before being fixed and visualized using DAPI (blue), α-tubulin (purple), total α-syn (red) or MJFR-14-6-4-2 α-syn antibody (green). Scale bar = 20 µm. Representative images of three biological replicates with 10 measurements in each experiment.* ***B)*** *Quantification of area of MJFR-14-6-4-2 α-syn signal relative to number of DAPI stained nuclei. Data are shown as mean of three independent experiments (n = 3, ***p < 0.001, one-way ANOVA followed by Tukey's multiple comparison test).*

Supplementary Figure 4

A) *Human α-syn expressing OLN-AS7 cells were exposed to PBS (Control) or 14 µg/mL (1 µM) sonicated α-syn PFFs prepared in PBS alone (α-syn PFFs) or in the presence of 2% DMSO (2% DMSO α-syn PFFs). After 24h of PFF treatment, the cells were washed to remove excess PFF and subsequently incubated for another 24h before being fixed and visualized using DAPI (blue), α-tubulin (purple), total α-syn (red) or anti-phospho-S129 α-syn (green). Scale bar = 20 µm. Representative images of three biological replicates with 10 measurements in each experiment.* ***B)*** *Quantification of area of anti-phospho-S129 α-syn signal relative to number of DAPI stained nuclei. Data are shown as mean of three independent experiments (n = 3, *p < 0.05, **p < 0.01, one-way ANOVA followed by Tukey's multiple comparison test).*

Supplementary Figure 5

SH-SY5Y ASYN cells were treated with dox to suppress α-syn expression (- α-syn) or relieved from dox treatment to induce α-syn overexpression (0% DMSO) for 7 days prior to fixation. To stop mitosis, the SH-SY5Y ASYN cells were treated with retinoic acid (10 µM final concentration) during the experiment. Quantification of area of MJFR-14-6-4-2 signal ***(A)*** *or anti-phospho-S129 α-syn signal* ***(B)*** *from individual coverslips relative to area of α-Tubulin signal (n = 4, with ~10 images for each condition in each experiment, **p < 0.01, ****p < 0.0001, student t-test).*

Supplementary Figure 6

Free floating 30 um sections of brain tissue from striatum or substantia nigra or paraffin embedded sections of duodenum and ileum from transgenic F28 mice, overexpressing human α-syn under the control of the endogenous mouse α-syn promoter. Twelwe transgenic F28 mice were divided into three groups with four in each and treated with water (0% DMSO), 10% DMSO (1 g/kg bodyweight) or 30% DMSO (3 g/kg bodyweight) for 14 days prior to sacrifizing and tissue collection. Striatum or substantia nigra sections from all animals were stained with an anti-tyrosine hydroxylase antibody and with the MJFR-14-6-4-2 antibody (n = 4 for each condition). The duodenum and ileum samples from all animals were stained with the MJFR14-6-4-2 antibody. n = 4-6 sections per segment (s2, s5, s8) were analyzed for the intestinal samples and one series of brain sections spanning the entirety of the brain from OB to beginning of cerebellum (30 µm sections, 10 series = 300 um distance between the sections for each series) for each animal. Scalebar = 20 µm for striatum, duedenum and ilium and 50 µm for substantia nigra.
